# Peripheral Nervous System Involvement in Late-Onset Cobalamin C Disease?

**DOI:** 10.3389/fneur.2020.594905

**Published:** 2020-11-26

**Authors:** Xujun Chu, Lingchao Meng, Wei Zhang, Jinjun Luo, Zhaoxia Wang, Yun Yuan

**Affiliations:** ^1^Department of Neurology, First Hospital, Peking University, Beijing, China; ^2^Department of Neurology, Temple University, Philadelphia, PA, United States

**Keywords:** cobalamin C disease, methylmalonic acid, homocysteine, peripheral neuropathy, MMACHC gene, MTHFR gene

## Abstract

**Background:** Cobalamin C (cblC) has a fundamental role in both central and peripheral nervous system function at any age. Neurologic manifestations may be the earliest and often the only manifestation of hereditary or acquired cblC defect. Peripheral neuropathy remains a classical but underdiagnosed complication of cblC defect, especially in late-onset cblC disease caused by mutations in the methylmalonic aciduria type C and homocysteinemia (*MMACHC*) gene. So the clinical, electrophysiological, and pathological characteristics of late-onset cblC disease are not well-known.

**Methods:** A retrospective study of patients with late-onset cblC disease was conducted at our hospital on a 3-year period. The neuropathy was confirmed by the nerve conduction study. Sural biopsies were performed in 2 patients.

**Results:** Eight patients were identified, with a mean onset age of 16.25 ± 6.07 years. All patients had methylmalonic aciduria, homocysteinemia, compound heterozygous *MMACHC* gene mutations were detected in all patients, and 7/8 patients with c.482G>A mutation. One patient concomitant with homozygote c.665C>T mutation in 5,10-methylenetetrahydrofolate reductase (*MTHFR*) gene. All patients showed limb weakness and cognitive impairment. Five patients had possible sensorimotor axonal polyneuropathy predominantly in the distal lower limbs. Sural biopsies showed loss of myelinated and unmyelinated fibers. Electro microscopy revealed crystalline-like inclusions bodies in Schwann cells and axonal degeneration.

**Conclusion:** Late-onset cblC disease had possible heterogeneous group of distal axonal neuropathy. c.482G>A mutation is a hot spot mutation in late-onset cblC disease.

## Introduction

The main causes of cobalamin deficiency are inadequate dietary intake (veganism, etc.), malabsorption (atrophic gastritis, parasitic Infection, chronic pancreatitis, pernicious anemia, etc.), and metabolic disturbance (transcobalamin gene polymorphisms, etc.) (1). Cobalamin C (cblC) disease is the most common inborn error of intracellular vitamin B12 metabolism caused by mutations in the methylmalonic aciduria type C and homocysteinemia (*MMACHC*) gene. It impairs conversion of dietary vitamin B12 and causes deficient synthesis of methylcobalamin and adenosylcobalamin. Diagnostic laboratory findings of cblC disease include elevated levels of methylmalonic acid in the urine, homocysteine in the blood (2–6), and serum propionylcarnitine (C3). Moreover, the ratios of C3-to-acetylcarnitine (C2) are remarkably elevated (6). Depending on the onset time of disease, the cblC disease can be divided into two types, early-onset type in the infancy period (2) and late-onset type in adolescents or adults (3–5).

The late-onset cblC disease is characterized by involvement of both the central nervous system and peripheral nerve system. The patients showed commonly cognitive and psychiatric disturbances (2, 3, 5–8), epilepsy (3–9), spastic paraplegia (3, 7, 8, 10, 11), with optic atrophy (3, 12). Some patients showed typical subacute combined degeneration (7, 13). Neuroimaging findings of the central nervous system were dominated by cerebral and cerebellar atrophy, with white matter hyperintensities (14) and spinal cord hyperintensities (13, 15).

Peripheral neuropathy remains a classical but underdiagnosed complication of cblC defect. Some reports indicated length dependent peripheral neuropathy (7, 8, 10). Nerve conduction study revealed reductions of conduction velocity or amplitude in the motor and sensory nerves (6–8, 14). Sural biopsy showed mixed neuropathy with demyelination and axonal degeneration in patients with central nerve involvement (7, 10). The objective of the present study was to verify and summarize the clinical, electrophysiological, and pathological features of peripheral nerve involvement in late-onset cblC disease.

## Materials and Methods

Clinical data from 8 unrelated patients with late-onset cblC disease who underwent peripheral nerve electrophysiological test in Peking University First Hospital from January 2016 to October 2019 were retrospectively analyzed (Table 1). The present study was approved by the institutional review board and ethics committee at Peking University First Hospital.

**Table 1:** Clinical manifestations in 8 late-onset cblC patients.

**No**.	**Gender**	**Age at diagnosis/onset year**	**Major symptoms**							**Major signs**		
			**Psychiatric changes**	**Coma**	**Clinical cognitive deficit**	**Limb weakness**	**Epilepsy**	**Vomiting**	**Anorexia**	**Pyramidal signs**	**Hyporeflexia**	**hypesthesia**
1	M	17/13	–	–	+	+	–	+	+	–	+	+
2	M	24/24	+	+	+	+	–	+	+	+	+	+
3	M	27/25	–	+	+	+	–	–	–	+	–	–
4	M	16/14	–	–	+	+	–	–	–	+	–	–
5	M	22/20	–	–	+	+	+	–	+	+	–	–
6	F	20/8	+	–	+	+	–	–	–	+	–	–
7	M	14/14	–	–	+	+	–	–	–	+	+	–
8	F	13/12	–	–	+	+	–	–	–	+	+	–

*+, positive result; –, negative result; cblC, cobalamin C; F, female; M, male*.

### Nerve Conduction Study

All patients were performed peripheral nerve conduction study (Alpine bioMed Aps corporation in Denmark), compound muscle action potentials (CMAPs) were recorded for the median nerve, the ulnar nerve, the tibial nerve, and the peroneal nerve with surface electrodes. Sensory nerve action potentials were recorded with surface electrodes from the median nerve, the ulnar nerve, and the sural nerve.

### Pathological Examination

Patient 1 and 2 were performed sural biopsies, part of sample was fixed in 4% formaldehyde, then paraffin-embedded, and sections were stained with Luxol fast blue, Congo red, hematoxylin, and eosin. The rest of each section was fixed in 2.5% glutaraldehyde then 1% buffered osmium tetroxide, followed by dehydrated and embedded in Epon. Semi-thin sections were stained with toluidine blue for light microscopy. Ultrathin sections were with uranyl acetate and lead citrate double-staining, then examined under an electron microscope.

## Results

### Clinical Data

All patients included 6 men and 2 women with body mass index ranging from 15.24 to 27.04 kg/m^2^. Of all 8 patients, the average age was 16.25 ± 6.07 years (13–27 years). The duration from onset to diagnostic time was 13.75 ± 12.71 months (2–35 months). Limb weakness (8/8) and cognitive impairment (8/8) were the most frequent clinical manifestations, followed by anorexia (3/8), psychiatric changes (2/8), vomiting (2/8), coma (2/8), numbness (1/8), and epilepsy (1/8). Additionally, 7 of 8 patients showed positive Babinski sign bilaterally in their lower limbs, hyporeflexia appeared in 4 of 8 patients, decreased vibration and pinprick sensations in lower limbs in 2 of 8 patients. Some patients presented with relapsing and remitting course. After 2–8 weeks treatments with intramuscular adenosylcobalamin (1 mg/d), oral L-carnitine (3 g/d), folic acid (5 mg/d), betaine (9 g/d) and compound vitamin B, all patients showed alleviation of clinical manifestations, the homocysteine and methylmalonic acid were in normal range.

### Laboratory and Imaging Findings

Laboratory investigations were remarkably elevated levels of urine methylmalonic acid and plasma homocysteine in 8 patients. Serum C3 levels were elevated in 3 patients, and the ratios of C3/C2 were elevated in 4 patients. Proteinuria was detected in 4 patients and hyperuricemia in 2. Cranial Magnetic resonance imaging revealed cerebral and cerebellar atrophy in 4 patients, ischemic infarcts in cerebral or basal ganglion in 2 patients (Table 2).

**Table 2:** Accessory examinations 8 late-onset cblC patients.

**No**.	**Methylmalonic aciduria (0.2–3.6) (μg)**	**Homocysteine (6–17) (μmol/L)**	**C3/C2 (0.03–0.50)**	**C3 (1.00–4.00) (μmol/L)**	**Cranial magnetic resonance imaging**
1	1012.83	93.20	1.00	2.73	Cerebral cortical atrophy
2	467.25	100.22	0.85	2.79	/
3	64.70	111.88	/	/	Cerebral cortical atrophy with lacunar infarcts
4	73.12	129.04	0.39	4.45	Normal
5	1213.92	230.97	0.78	6.81	Cerebral cortical atrophy
6	687.99	219.72	0.49	5.12	Normal
7	190.10	72.37	0.85	4.17	Cerebral cortical atrophy
8	41.66	86.56	0.18	4.70	Normal

*cblC, cobalamin C; C3, propionylcarnitine; C2, acetylcarnitine; /, not tested*.

### Mutation Analysis

Next generation sequencing revealed all patients with compound heterozygous mutations in the *MMACHC* gene. A missense mutation of c. 482G > A (VCV000001425.8) was detected in patient 1–5, 7, and 8. A deletion mutation of c.658_660delAAG (VCV000095707.7) was detected in patient 1, 6. A nonsense mutation of c.217C>T (VCV000203825.5) in patient 2, a frameshift mutation of c.440_441del (VCV000203834.4) in patient 3, a duplication mutation of c.271dupA (VCV000001421.17) in patient 8. A nonsense mutation of c.609G>A (VCV000030800.6) was detected in patient 4, 5, and 7. A missense mutation of c.347T>C (VCV000001422.3) was detected in patient 6. The parents of patient 1, 3, 4, 7, and 8 were confirmed to be heterozygous carriers of the *MMACHC* gene mutations, while patient 2, 5, and 6 did not perform pedigree verification. Finally, Patient 1 further showed homozygous missense mutations of c.665C>T (VCV000003520.13) in 5,10-methylenetetrahydrofolate reductase (*MTHFR*) gene, whose parents were heterozygous carriers of the *MTHFR* gene mutations.

### Nerve Conduction Study

Nerve conduction study (Table 3) revealed decreased motor nerve conduction velocities with reduced amplitude of CMAPs of common peroneal nerve and tibial nerve in patient 1 and 6. Reduction of motor nerve conduction velocities with reduced amplitude of CMAPs and prolonged distal motor latencies in bilateral tibial nerve and prolonged distal motor latencies of bilateral common peroneal nerve, absence of the sensory nerve action potentials amplitude in the bilateral posterior tibial nerve, superficial peroneal nerve, were recorded in patient 2. Reduction of sensory nerve conduction velocities of the right sural nerve, the prolonged distal motor latencies and decreased amplitude of the CMAPs and reduction of motor nerve conduction velocities of the right tibial nerve and the left common peroneal nerve were recorded in patient 8. Decreased amplitude of Sensory nerve action potentials of right sural nerve and decreased amplitude of CMAPs of left common peroneal nerve were recorded in patient 7. No obvious abnormality was found in the nerve conduction study of patient 3 and 5. The somatosensory evoked potentials P30 and P38, and the bilateral visual evoked potentials P100 were not recorded in patient 3 and 5. The T10-L4 evoked potentials of spinal cord conduction velocity in patient 3 were not recorded. The somatosensory evoked potential N9 was not recorded in patient 4. No sympathetic skin response in the right lower limb and prolonged latency of the left lower limb were recorded in patient 6. The sympathetic skin response was prolonged in both upper limbs and lower limbs in patient 4.

**Table 3:** Electrophysiological data in 5 late-onset cblC patients.

**Motor/sensory**	**Nerve**	**No**.	**Left dL (ms)**	**Left amplitude (mV/uV)**	**Left conduction velocities (m/s)**	**Right dL (ms)**	**Right amplitude (mV/uV)**	**Right conduction velocities (m/s)**
Motor nerve	Median nerve	1	3.48	14.40	57.60	3.20	12.30	56.60
		2	3.40	9.40	64.00	/	/	/
		6	3.00	15.50	57.50	3.00	16.20	56.10
		7	2.90	14.30	53.30	/	/	/
		8	3.20	13.80	60.50	/	/	/
	Ulnar nerve	7	2.90	5.40	55.10	/	/	/
		8	2.20	10.00	51.00	/	/	/
	Peroneal nerve	1	5.25	1.19	33.20	5.80	1.12	30.50
		2	6.35	2.60	41.30	5.00	4.10	40.10
		6	3.23	2.40	37.60	3.41	3.00	37.70
		7	5.00	2.10	45.80	/	/	/
		8	5.60	5.10	40.50	/	/	/
	Tibial nerve	1	7.48	0.76	31.70	5.67	0.35	28.10
		2	6.28	4.00	37.70	7.96	3.90	35.90
		6	3.58	8.40	38.40	4.41	7.30	38.20
		7	/	/	/	5.20	4.60	44.60
		8	/	/	/	6.90	0.60	39.20
Sensory nerve	Median nerve	1	2.68	15.60	54.10	2.85	13.10	51.90
		2	3.30	13.90	51.10	/	/	/
		6	2.81	15.20	49.80	2.96	16.30	48.00
		7	2.10	11.00	50.00	/	/	/
		8	2.00	25.00	43.50	/	/	/
	Ulnar nerve	1	2.44	14.60	47.10	2.50	10.40	48.00
		2	3.01	12.30	29.90	/	/	/
		6	2.23	6.00	42.60	2.62	3.30	43.90
	Superficial peroneal nerve	1	3.03	12.30	42.90	–	–	–
		2	–	–	–	–	–	–
		6	2.10	14.80	42.90	2.52	13.00	41.70
	Posterior tibial nerve	1	2.88	6.40	43.40	2.47	6.50	46.60
		2	/	/	/	–	–	–
		6	2.05	8.40	46.30	2.08	5.00	48.10
	Sural nerve	7	/	/	/	2.20	11.00	50.00
		8	/	/	/	2.30	25.00	43.50

–*, no response; /, not tested; cblC, cobalamin C; dL, distal latency*.

### Nerve Pathology

**Figure 1:**
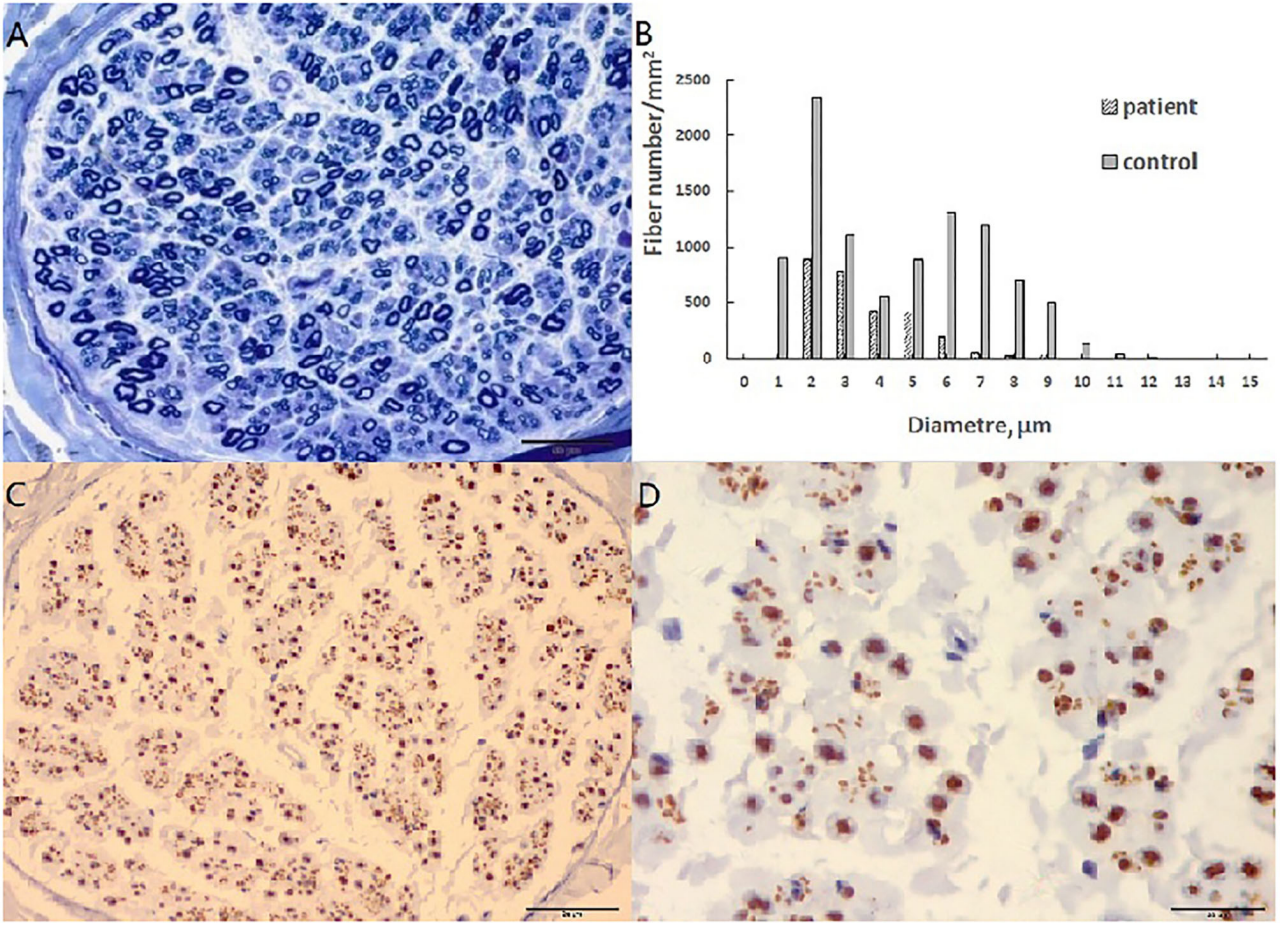
Sural biopsy in cblC disease. **(A)** Diffuse loss of myelinated fibers, predominant the large myelinated fibers. **(B)** Histograms of myelinated fibers, the entity is the control, and the slash is the patient with loss of large myelinated fibers. **(C)** Moderate loss of axons with different diameter. **(D)** High magnificent showed unmyelinated fibers loosely distributed without a normal cluster profile. Scale bar = 50 μm in **(A,C)**. Scale bar = 20 μm in **(D)**.

**Figure 2:**
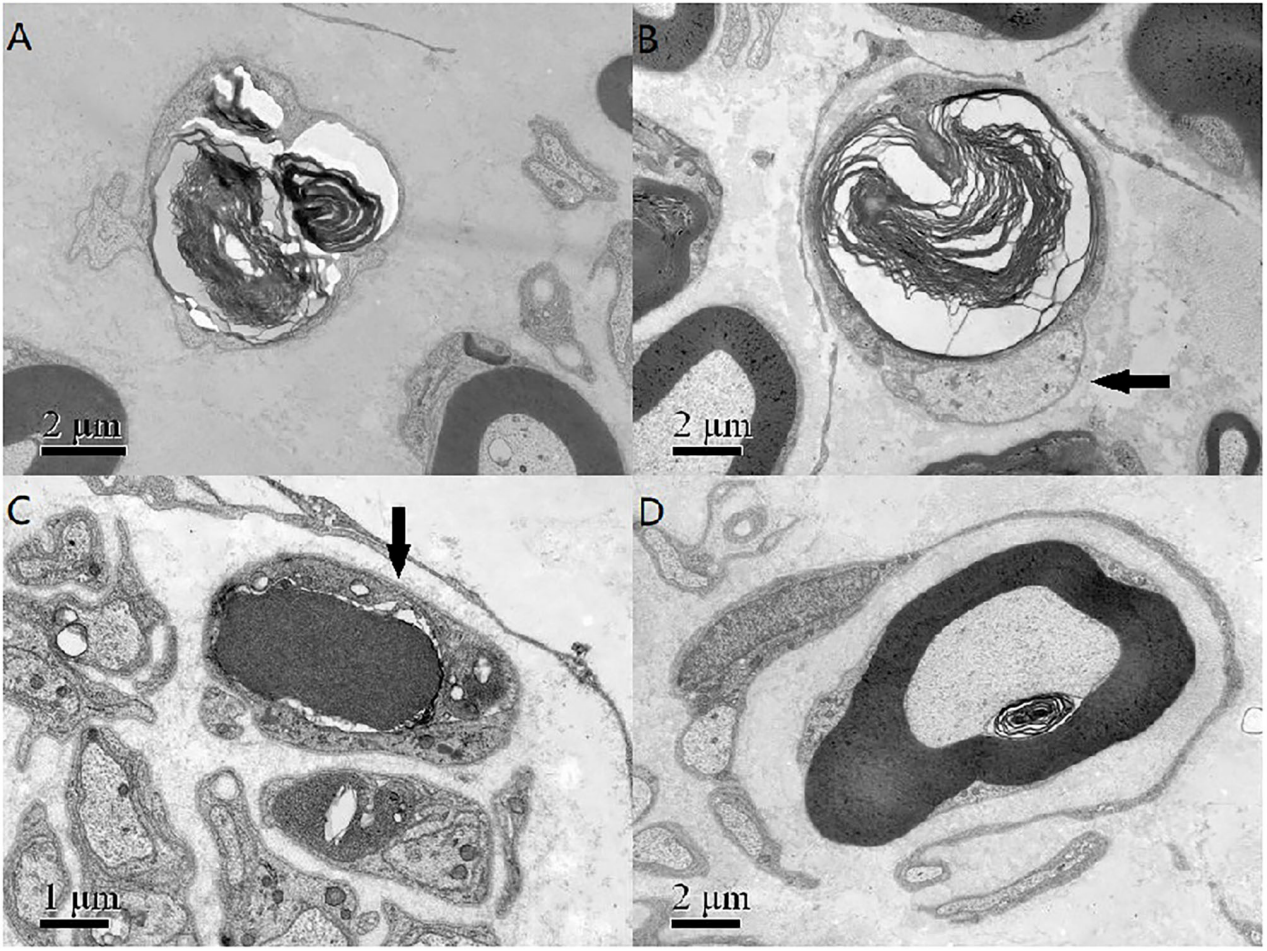
**(A)** The axonal degeneration characterized by myelin remnants with irregular laminated structure within Schwann cells. **(B)** Myelin remnants with circular arrangement of myelin debris with an axon (arrow) surrounded by Schwann cell cytoplasm. **(C)** There was a non-membranous bounded osmiophilic dense crystalline-like inclusion bodies in a Schwann cell (arrow), and an osmiophilic inclusion with a fissure and organelle in another Schwann cells. **(D)** The Schwann cell processes and unmyelinated nerve fibers around a myelinated fibers formed an atypical onion bulb structure. Scale bar = 2 μm in **(A,B,D)**. Scale bar = 1 μm in **(C)**.

A pathological examination was conducted on two patients who showed moderate loss of myelinated fibers (Figure 1A). The density of myelinated fiber was 2,787/mm^2^ (9,663/mm^2^ in the control), with predominantly loss of large myelinated fibers (Figure 1B). Immunohistochemical staining with an anti-neurofilament antibody showed severe loss of both myelinated fibers and unmyelinated fibers (Figure 1C). The rest unmyelinated fibers loosely distributed, without a normal cluster profile (Figure 1D). Additionally, clusters of regenerating myelinated fibers, degeneration of myelinated fibers and atypical onion bulb formations were observed. Ultrastructural assessment further confirmed axonal degeneration with regenerated clusters (Figure 2A). The axonal degeneration characterized by myelin remnants with irregular laminated structure within Schwann cells. Frequently, some myelin remnants with irregular circular laminated structure nearby an abnormal axon surrounded by Schwann cell cytoplasm (Figure 2B). The axons are filled with fine fibrillar material. Some Schwann cells contain crystalline-like inclusions bodies (Figure 2C). Occasionally, Schwann cell processes were arranged myelinated fibers in circular configurations (Figure 2D). The collagen pocket was observed generally.

## Discussion

All patients in present series showed adolescence onset, similar to reported patients in China (3, 6). The main symptoms were involvement of central nervous system, similar to other reports (3, 6, 8, 16). The gastrointestinal disorders appeared also frequently in our cohort, which were rarely reported among late-onset patients (3). Cranial magnetic resonance imaging revealed cerebral atrophy with lacunar infarcts in our patients and also other reports (3, 14). All patients had high levels of urine methylmalonic acid and the plasma homocysteine. Compound heterozygous mutations in the *MMACHC* gene were identified and confirmed the diagnosis of late-onset cblC disease in them.

Sensorimotor peripheral demyelinating neuropathy appeared in early-onset methylmalonic aciduria and homocystinuria, cblC type (17). We found that peripheral nerve involved in late-onset cblC disease, the peripheral nerve involvement was a predominant manifestation in one patient without pyramidal signs, the most symptoms other patients showed were mild sensory or autonomic symptoms, combined with pyramidal signs (3, 14, 18). Like our patient with multivitamin therapy, relapsing and remitting myelopathy and neuropathy as main symptoms were also described in late-onset cblC disease (10). The vitamin B12 responsive neuropathy should systematically be ruled out in the clinical setting of sensory neuropathy or idiopathic neuronopathy because of potential reversibility (1).

The neurophysiological studies in present series indicated that 75% patients presented with peripheral nerve involvement, similar to other report with 83% patients presented with mild to moderate peripheral nerve damage (6). We also found that the neurophysiological findings varied largely due to the severity variation in different patients. Some patients showed mixed axonal and myelinated neuropathies in both sensory and motor nerves, usually intermediate conduction velocity with reduction of amplitudes (7, 14). We found that some patients showed possible axonal neuropathy with a decrease in the amplitudes of sensory and motor nerves, similar to a recent case report (19). Decreased conduction velocity was less commonly detected also in other reported late-onset cblC disease (6, 14). Similar results appeared in other vitamin B12 responsive neuropathy (1, 20).

A sural biopsy specimen displayed general axonal degeneration with demyelination in the peripheral nerves in late-onset cblC disease. Both demyelination and axonal degeneration in peripheral nerves were described in other report (7). In addition of regeneration cluster, we found axonal degeneration characterized by myelin remnants with irregular laminated structure within Schwann cells. Some myelin remnants with circular arrangement of myelin debris with abnormal axon surrounded by Schwann cell cytoplasm (Figure 2B) which might be a specific form of axonal degeneration rather than a demyelination process. Some Schwann cells had crystalline-like inclusions bodies, which were also described as Fardeau-Engel bodies in other neuropathy (21). Appearance of atypical onion ball structure indicated a mininal demyelination which was described in axonal CMT (22). Moreover, we found the loss of unmyelinated fibers in late-onset cblC disease, which was described also in vitamin B12 deficiency (23).

We confirmed that c.482G>A mutation in *MMACHC* gene was the hot spot mutation (3, 6, 24). Patients with c.482G>A mutation had late-onset symptoms and easier metabolic control (25). A reported late-onset patient with mutations (c.217C>T and c.482G>A) same as our patient 2 showed more severer peripheral nerve impairment than patient 2 (19). Peripheral nerves were more frequently identified in late-onset type with point mutation (3). We found that peripheral nerve impairment was most severe in the patient concomitant with a homozygous c.665C>T mutation in *MTHFR* gene. Elevated homocysteine could have contributed to increased peripheral nerve damage (26). However, given our limited data, it is difficult to determine the linear relationship between homocysteine and peripheral nerve damage.

In fact, only one patient presented with typical peripheral neuropathy, and 5 patients showed subclinical peripheral nervous system involvement. It is difficult to identify the levels of the lesions such as limb weakness concerning the central nervous system as spinal cord or the peripheral nervous system or both, our electrophysiological data and pathology findings were not very specific and significant of peripheral nerve lesions, the examinations provided the possible evidences of the peripheral nervous system involvement. We need to follow patients in a long time to re-evaluate levels of the peripheral nerve lesions.

In summary, our study highlights peripheral nervous system involvement in late-onset cblC disease. Pathological findings in our patients reveal new insights that possible distal axonal neuropathy in cblC patients cannot be ignored.

## Data Availability Statement

The original contributions presented in the study are included in the article/Supplementary Materials, further inquiries can be directed to the corresponding author.

## Ethics Statement

Written informed consent was obtained from the individual(s) for the publication of any potentially identifiable images or data included in this article.

## Author Contributions

XC and YY contributed to the conception and design of the study. XC collected and organized the database. XC wrote the first draft of the manuscript. LM and WZ wrote sections of the manuscript. ZW and JL participated in the design. All authors contributed to manuscript revision, read, and approved the submitted version.

## Conflict of Interest

The authors declare that the research was conducted in the absence of any commercial or financial relationships that could be construed as a potential conflict of interest.
