# Supplementary material for: Peripheral Nervous System Involvement in Late-Onset Cobalamin C Disease?
Source: Front Neurol. 2020 Nov 26;11:594905. doi: 10.3389/fneur.2020.594905 (PMC7726435; doi:10.3389/fneur.2020.594905)
Supplement: Supplementary file 1 [file Data_Sheet_1.PDF]

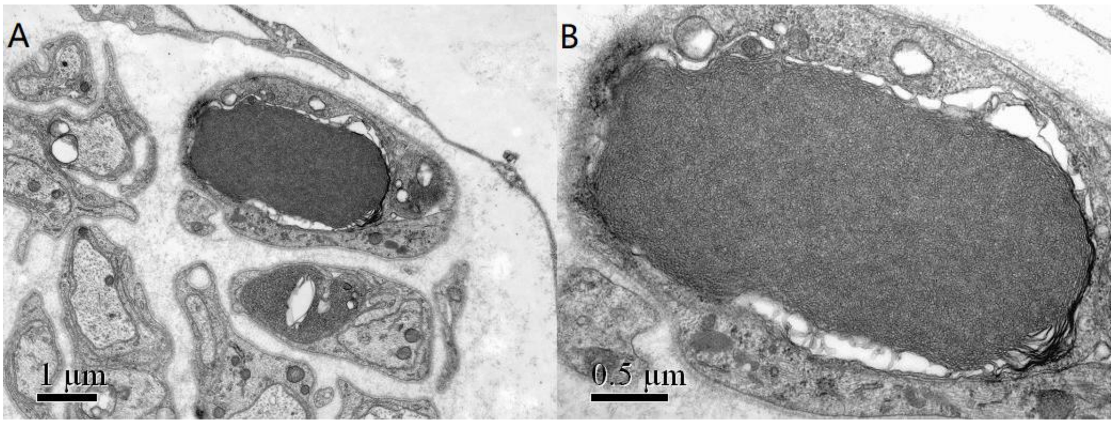

Figure S1. (**A**) There was a non-membranous bounded osmiophilic dense crystalline-like inclusion bodies in a Schwann cell, and an osmiophilic inclusion with a fissure and organelle in another Schwann cells. (**B**) High magnification showed lamellar structure in the peripheral area of the osmiophilic dense inclusion.

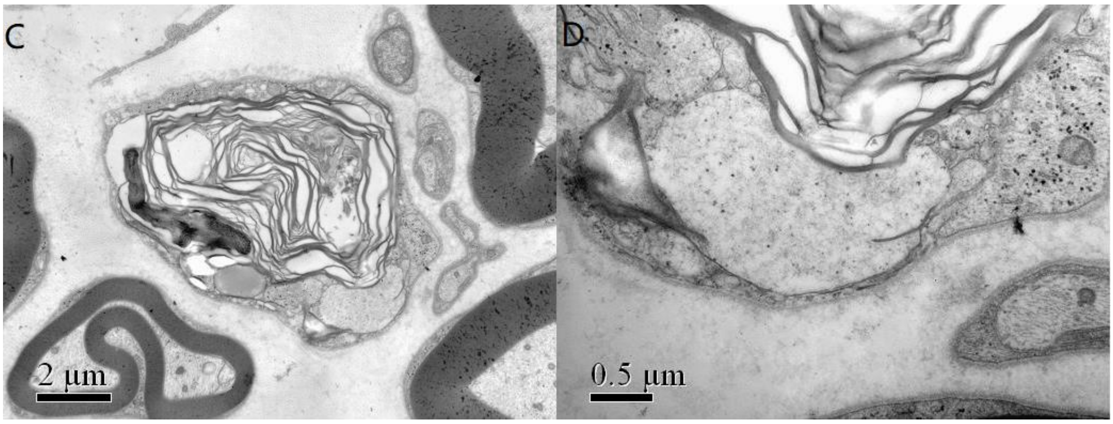

Fig S2. (**C**) Myelin remnants with circular arrangement of myelin debris with an axon surrounded by Schwann cell cytoplasm. (**D**) Another myelin remnant with high magnification of axon which filled fine fibrillar material.
